# Contamination and health risk assessment of lead, arsenic, cadmium, and aluminum from a total diet study of Jilin Province, China

**DOI:** 10.1002/fsn3.1851

**Published:** 2020-09-04

**Authors:** Bo Wang, Ya Liu, Hui Wang, Lianzhi Cui, Zhihao Zhang, Jinzhi Guo, Sijie Liu, Weiwei Cui

**Affiliations:** ^1^ Department of Nutrition and Food Hygiene School of Public Health Jilin University Changchun China; ^2^ Department of Physical and Chemical Test Jilin Provincial Center for Disease Control and Prevention Changchun China; ^3^ Department of Clinical Laboratory Jilin Cancer Hospital Changchun China

**Keywords:** aluminum, arsenic, cadmium, heavy metals, lead, total diet study

## Abstract

Lead (Pb), arsenic (As), cadmium (Cd) and aluminum (Al) are the four most common heavy metals and can cause serious harm to human health. To evaluate contamination levels and associated safety issues of the four common heavy metals of the residents in Jilin Province, China, a total diet study (TDS) method was used. Concentration and consumption data of the four heavy metals were collected from the fifth Chinese TDS of Jilin province. In total, 12 food groups were studied and two regions were selected for comparison. According to the results, the mean concentration of lead, arsenic, cadmium, and aluminum was 0.0189, 0.0691, 0.0085, and 9.309 mg/kg, respectively. Aluminum in deep‐fried dough sticks exceeded the national limit standard. Pollution of the Songhua River Basin is not very different from that of other areas. The average consumer exposure to the four heavy metals in the 2 to 6‐year‐old group was the highest among all age‐groups. Potatoes and their products were the primary sources of dietary exposure to lead. Aquatic products and their related commodities had the highest contribution to arsenic exposure. Vegetables and vegetable products were the main sources of dietary exposure to cadmium. The highest contributor to aluminum in the diet was from cereals and cereal products. In general, there might be some potential risks to the 2 to 6‐year‐old population due to exposure to lead and aluminum. Contamination of aluminum in cereals and cereal products needs further consideration.

## Graphical abstract

This study evaluated contamination levels and associated safety issues of four common heavy metals, lead, arsenic, cadmium, and aluminum, using a total diet study of people from Jilin Province, China. The exposures to lead, arsenic, cadmium, and aluminum through the diet are at relatively safe levels; however, there might be some potential risks to the 2‐ to 6‐year‐old population due to exposure to lead and aluminum.

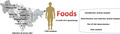

## 1 INTRODUCTION

With the rapid development of China's economy, environmental pollution and food chain pollution have become a social concern, of which heavy metal pollution is the most serious. Dietary intake is the main way for humans to ingest heavy metals. Long‐term consumption of foods containing heavy metals will greatly increase the risk of exposure to heavy metals and adverse effects on human health. As a result, the residual amount of pollutants in food and their intake levels have attracted worldwide attention. Among them, heavy metal pollution in food is one of the food safety issues that people focus on (Cheng, 2003). Research has shown that the hazards of heavy metal pollution to human health are serious (Ochola and Masibo, 2014). Heavy metals such as, lead (Pb), arsenic (As), cadmium (Cd), and aluminum (Al) can cause potential harm to human health. Pb is associated with decreased intelligence quotient (IQ) in children and increased systolic blood pressure in adults (EFSA Journal, 2010). Cd can cause kidney damage (OSTI.GOV, 1989). The United States Environmental Protection Agency and the International Agency for Research on Cancer both regard As to be a class I carcinogen in humans and believe that As is associated with lung cancer (Joseph & Mcbean, 2015). Al can affect the reproductive and developing nervous system and was also recognized to be a potential neurotoxin in humans and animals (EFSA Journal, 2007a). It has been shown that Al inhibits the activity of acetylcholinesterase in the brain (Moraes & Leite, 1994) and causes increased free radical effects (El‐Demerdash, 2005). Heavy metal contamination in diet is a common food safety concern worldwide.

Jilin Province is a large agricultural province with few heavy metal polluting industries, which makes the degree of heavy metal pollution in Jilin Province lighter than in other industrially developed areas. However, as a typical region of the old industrial base in northeast China, Jilin's industry has recovered and been redeveloped; at the same time, the environment has gradually deteriorated. This is especially true in the Songhua River basin, including Changchun, Jilin, Songyuan, Baicheng, and some of the subordinate areas, where there are many agricultural processing factories and chemical factories discharging heavy metals (Wu, Liang, & Tian, 2012). Thus, there may be some risk of dietary heavy metal exposure to residents around these areas. Therefore, three regions from the Songhua River basin were selected to study the dietary heavy metal exposure of their residents.

Three methods are used to evaluate dietary chemical pollutants and the nutrient intake of residents in a country or region: single‐food selectivity study, double‐meal study, and total diet study (TDS), which is also known as a market basket survey and is an internationally recognized evaluation of a country by directly detecting the contents of various pollutants in all meals (including drinking water) cooked by residents to obtain the dietary intake of food pollutants in a population or subpopulation. The measured food samples are close to people's actual consumption status. This method is considered to be a general and leading method to evaluate the intake of chemical pollutants and nutrients in the diet of a large‐scale population in a region or county (Joint FAO & WHO, 1985; World Health Organization, 2005). In this study, the total dietary research method was used to assess the dietary intake of heavy metals for residents of Jilin Province.

## 2 MATERIALS AND METHODS

### 2.1 Dietary survey

#### 2.1.1 Selection of survey sites

According to the principle of multistage sampling, the samples represent the totality. The principle of selecting survey points is that rural areas should be chosen from areas that represent the dietary habits and medium‐sized economic conditions of rural residents in the province, while urban areas should be chosen from small‐ and medium‐sized cities. The general principle is to take full account of the survey points selected to represent the dietary habits, nutritional status, and dietary structure of residents in the province. At the same time, it also combines the data of the local residents' economic situation surveyed by the statistical department. It is required that the comprehensive results obtained from the six selected survey sites represent the average dietary composition and level of residents in the province (Qiu, Lyu, Zhou, Zhao, & Wu, 2017; Zhang, Gao, & Li, 2008). In Jilin Province, it is a specific implementation unit (a small vegetable basket). There are six survey points, namely Siping City, Ningjiang District of Songyuan City (two urban points), Yushu City (county‐level city), Huadian City (county‐level city), Dongfeng County, and Tonghua County (four rural points).Siping is a representative of a small‐ to medium‐sized city and Dongfeng County and Tonghua County are representatives of the rural points in the non‐Songhua River area. In addition, the Ningjiang District of Songyuan City represents small‐ and medium‐sized cities, and Yushu City (county‐level city) and Huadian City (county‐level city) represent rural points in the Songhua River area.

#### 2.1.2 Selection of foods

Consumption data were from the sixth China national total diet survey, which was during 2015 and 2016. The consumption data in this study included 7,700 residents in Jilin Province, China. The dietary survey was conducted among all family members in an individual unit. The methods involved 3 days with 24‐hr questioning, and registration was adopted. The survey covered all food and drinking water consumed by households or subjects during the survey, as well as the variety, quantity, and use of all condiments. Individual dietary surveys were calculated according to their gender and age. The daily consumption of various foods per capita of different age‐groups in the province was calculated by the weighted average statistical method.

#### 2.1.3 Classification of food samples

According to the mixed food sample method currently used, the per capita food consumption was divided into 12 categories and/or 48 product groups. The selected food groups included cereals and cereal products; beans, nuts, and their products; potatoes and their products; meat and its products; eggs and egg products; aquatic products and their commodities; milk and dairy products; vegetables and vegetable products; fruits and fruit products; sugar and sugar products; drinking water and beverages; and alcoholic drinks. Through this process, the dietary composition of the investigated area or population was obtained.

### 2.2 Sample collection and processing

#### 2.2.1 Determination and collection of food samples

According to the results of the consumption data, the individual food consumption was clustered, and the daily consumption of all kinds of food was calculated for residents of Jilin Province according to different age‐groups. The representative food samples determined according to the clustering results were collected at the neighborhood committees or nearby food purchase points in accordance with the sampling procedure.

#### 2.2.2 Preparation of food samples

Collected samples were cooked according to local cooking methods and cooking utensils. The cooked food samples were crushed and homogenized, and then put into high‐pressure polyethylene plastic containers and frozen in a low‐temperature refrigerator at −20°C.

### 2.3 Sample determination method

#### 2.3.1 Reagents

All solutions used in this study were prepared with analytical‐grade chemicals, and the ultrapure water (18.25 Ω) for laboratory use was generated by a Milli‐Q IQ 7000 Purification System (Millipore).

- 5% nitric acid, serial number 5183–4687, batch number 26‐081CRY2, was purchased from Beijing Microelectronics Factory.
- Environmental mixed standard solutions: Solutions containing 100 mg/L of each element (Ag, Al, As, Ba, Be, Cd, Co, Cr, Cu, Mn, Mo, Ni, Pb, Sb, Se, Tl, V, Zn, and U) were purchased from Agilent Corporation. Working standards in 2% HNO_3_ were prepared daily.
- Internal standard solution: The internal standard liquid was In (National Iron and Steel Material Testing Center, 1,000 µg/ml, GSBG 62041‐90 lot number 4901).
- Mixed tuning fluid: The tuning fluid was used to optimize the performance of inductively coupled plasma mass spectrometry (ICP‐MS) before use. A 10 mg/L multi‐element solution (Thermo Elemental), batch number 1009, was used. This contained lithium (Li), cobalt (Co), indium (In), and uranium (U) and was capable of covering the wide range of masses that were needed.

#### 2.3.2 Sample digestion procedure

Accurately weighed solid samples of 0.3–0.5 g and liquid samples of 1.0 g (accurate to 0.0001 g) were added to 5 ml HNO_3_ in a high‐pressure digestion tank; a blank experiment was also run. Catchup acidification was performed at 100°C on an acid catcher to exhaust the large amount of yellow smoke. Next, we wrapped the external tank, digested at 100°C for 2 hr, digested at 140°C for 2 hr and finally, digested at 160°C for 2 hr. After digestion, the digestive solution was driven to less than 1 ml at 100°C on the acid catcher. After cooling to room temperature, the digested solutions were completed with ultrapure water to the final volume before analysis.

#### 2.3.3 The ICP‐MS determination procedure

A Thermo X‐Series 2 ICP‐MS with a Supplementary Automatic Sampling Device (Thermo) was used. Two peristaltic pumps were used to pump the sample solution (including blank solution, standard solution, sample solution, and internal standard solution). The working parameters of ICP‐MS were as follows: RF power: 1,200 W; proton channel number: 3; carrier gas flow: 0.91 L/min; sample extraction time: 30 s; sampling cone depth: 120 mm; four‐stage bar partial pressure: 0.89 V; sampling cone level: 87 mm; six‐stage bar bias V: −2.4; sampling cone vertical: 119 mm; scanning step: 150; measurement mode: peak jump, and focus: 6.0.

#### 2.3.4 Calibration

The standard series of elements with concentrations of 0.0, 10.0, 50.0, 100.0, 200.0, and 500.0 ng/ml were prepared by individually diluting the mixed standard solution with 2% HNO_3_. The experimental results showed that the linear relationship of elements in the above ranges was good, and the correlation coefficient was >0.999.

#### 2.3.5 Quality control

The certified reference material GBW 10011 was used to determine the metal content in wheat flour by ICP‐MS. The standard deviation was 4.51%–9.44%, which met the needs of food physical and chemical detection (Table 1).

**TABLE 1:** Determination of heavy metals in wheat flour reference material

Element	Measured value (mg/kg)				Standard reference value (mg/kg)	RSD (%)
	1	2	3	4		
Al (27)	94.3	111.1	112.4	101.3	104 + 10	9.44
As (75)	0.0029	0.0031	0.0035	0.0029	0.031 + 0.005	9.12
Cd (111)	0.018	0.017	0.019	0.018	0.018 + 0.004	4.54
Pb (208)	0.072	0.059	0.068	0.061	0.065 + 0.024	9.32

### 2.4 Evaluation method

#### 2.4.1 Heavy metal contamination evaluation

Determination of Pb, As, and Cd contamination in food samples was performed according to the National Food Safety Standard (GB 2762‐2017). Determination of Al contamination in food samples was in accordance with the National Food Safety Standard (GB 2760‐2014).

#### 2.4.2 Method for calculating residents' total dietary exposure

To assess total dietary exposure in Jilin Province to the four heavy metals, we used the point evaluation model. The formula was

$$Exp_{i}=\sum_{t=1}^{n}\frac{F_{i}\times C_{i}}{w}$$

where EXP*_i_* was daily exposure to a certain heavy metal for an individual, with the units μg/kg bw/day. *F_i_* was the mean consumption data of food*_i_*, with the units g/day. *C_i_* was the mean concentration of a certain heavy metal in food*_i_*. *W* was mean weight of each individual age‐group.

The formula for calculating the contribution rate of a certain kind of food was: Dietary contribution rate of certain foods (%) = exposure of each heavy metal to certain foods ÷ the sum of all kinds of food exposure × 100%.

#### 2.4.3 Risk characterization

According to the safety protection principle of risk assessment, the content of total As instead of inorganic As was evaluated in this paper. According to the Joint FAO/WHO Expert Committee on Food Additives (JECFA) evaluation report, there is no clear health guidance value for describing the risk characteristics of inorganic As and Pb. In this study, the exposure boundary (MOE) method was used to assess the risk of inorganic As and Pb exposure in the total diet of residents in Jilin Province. Thus, the greater the ratio of the inorganic As or Pb baseline dose to the corresponding exposure dose, the greater the MOE value. For a lower risk of exposure, the lower limit of the benchmark dose (BDML 0.5) with inorganic As‐induced lung cancer as the end point of toxicity effect was 3–5 µg/kg BW (Joint FAO/WHO Expert Committee on Food Additives & WHO Organization, 2011b). According to the safety protection principle of risk assessment, 3 µg/kg BW was taken as the BDML_0.5_ value in this study. For Pb, the BMDL_01_ for adults was 1.2 µg/kg BW/day (systolic blood pressure increased by 1 mmHg) and for children was 0.6 µg/kg BW/day (IQ decreased by 1 IQ point) (Joint FAO/WHO Expert Committee on Food Additives & WHO Organization, 2011c).

The provisional tolerable monthly intake (PTMI) of Cd in food formulated by JECFA at its 73rd meeting in 2011 was 25 µg/kg BW (equivalent to 0.833 µg/kg BW per day) (WHO, 2011a). The provisional tolerable weekly intake (PTWI) of Al in food determined by JECFA at its 74th meeting in 2011 was 2 mg/kg BW (equivalent to 0.286 mg/kg BW per day) (WHO, 2011b). Comparing the exposure of Cd and Al with the corresponding health guidance value, a lower exposure value indicated that the risk was acceptable.

#### 2.4.4 Data analysis

WHO recommends that for data less than the limit of detection (LOD), if not more than 60% of the data is less than LOD, these data should be calculated according to 1/2 LOD. In this study, the four heavy metal contents of samples less than LOD were calculated according to 1/2 LOD. Because of the strong toxicity of inorganic As, the safety of As was evaluated based on inorganic As in the world; inorganic As accounted for approximately 70% of the total As in food (Joint FAO/WHO Expert Committee on Food Additives & WHO Organization, 2011b). In this study, total As was evaluated as inorganic As. Data processing and statistical analysis were completed using Excel 2007 and IBM SPSS 17.0 software.

## 3 RESULTS

### 3.1 Contamination of the four heavy metals in total diet

Tables 2 and 3 show the mean concentration of the four heavy metals in the total diet of Jilin Province and contamination levels in different basins. The results showed that pollution of the Songhua River Basin was not very different from that of other areas. For Pb, the food product categories with the highest concentration were potatoes and their products (0.0610 mg/kg); among the categories, the highest concentration was vermicelli (0.0697 mg/kg). For As, the food product categories with the highest concentration were aquatic products and their derivatives (0.728 mg/kg); among the categories, the highest concentration was cod (1.403 mg/kg). For Cd, the food product categories with the highest concentration were beans, nuts, and their products (0.0240 mg/kg); among the categories, the highest concentration was peanut (0.0841 mg/kg). For Al, the food product categories with the highest concentration were cereals and cereal products (44.016 mg/kg); among the categories, the highest concentration was deep‐fried dough sticks (250.120 mg/kg). The Ministry of Health of China recommends that the maximum allowable concentration (MAC) for Al in fried cereals is 100 mg/kg; the deep‐fried dough sticks were above the MAC.

**TABLE 2:** Mean concentration of Pb, As, Cd, and Al in the total diet of Jilin Province, China

Food group	Product group	Pb, mg/kg			As, mg/kg			Cd, mg/kg			Al, mg/kg		
		Mean	Songhua River Area	Other Area	Mean	Songhua River Area	Other Area	Mean	Songhua River Area	Other Area	Mean	Songhua River Area	Other Area
Cereals and cereal products	Noodles	0.0136	0.0214	0.0058	0.0245	0.0247	0.0243	0.0030	0.0030	0.0030	3.5444	4.2000	2.8888
	Steamed buns	0.0151	0.0191	0.0112	0.0227	0.0231	0.0222	0.0048	0.0030	0.0066	18.4118	6.0886	30.7350
	Deep‐fried dough sticks	0.0044	0.0052	0.0035	0.0236	0.0185	0.0286	0.0047	0.0030	0.0065	228.3606	206.6015	250.1196
	Rice	0.0041	0.0048	0.0035	0.0400	0.0364	0.0435	0.0030	0.0030	0.0030	5.3961	7.7848	3.0073
	Corn	0.0047	0.0059	0.0035	0.0141	0.0157	0.0124	0.0030	0.0030	0.0030	3.5938	4.4709	2.7167
	Bread	0.0035	0.0035	0.0035	0.0202	0.0249	0.0154	0.0065	0.0061	0.0070	4.7900	3.6284	5.9515
Beans, nuts, and their products	Soybean	0.0501	0.0581	0.0421	0.0189	0.0155	0.0224	0.0113	0.0110	0.0115	4.1434	4.2628	4.0240
	Bean curd	0.0557	0.0520	0.0593	0.0271	0.0221	0.0321	0.0104	0.0084	0.0123	4.9167	4.3405	5.4930
	Soybean milk	0.0239	0.0161	0.0317	0.0068	0.0040	0.0095	0.0030	0.0030	0.0030	1.1945	1.4779	0.9111
	Dried bean curd	0.0540	0.0436	0.0643	0.0270	0.0206	0.0334	0.0113	0.0130	0.0096	3.9978	4.5061	3.4895
	Peanut	0.0403	0.0449	0.0357	0.0188	0.0212	0.0163	0.0841	0.0489	0.1193	3.1473	2.6363	3.6582
Potatoes and their products	Potato	0.0523	0.0573	0.0473	0.0140	0.0160	0.0120	0.0211	0.0219	0.0204	3.8394	4.2599	3.4188
	Vermicelli	0.0697	0.0603	0.0791	0.0074	0.0108	0.0040	0.0030	0.0030	0.0030	29.0077	30.8536	27.1618
Meat and its products	Pork	0.0385	0.0364	0.0406	0.0234	0.0243	0.0224	0.0030	0.0030	0.0030	2.7164	2.3849	3.0479
	Pork liver	0.0410	0.0386	0.0434	0.0113	0.0087	0.0139	0.0285	0.0265	0.0305	3.7661	3.5078	4.0244
	Sausage	0.0361	0.0315	0.0406	0.0088	0.0040	0.0135	0.0030	0.0030	0.0030	3.5803	3.0860	4.0747
	Beef	0.0367	0.0448	0.0287	0.0249	0.0280	0.0217	0.0030	0.0030	0.0030	7.1642	11.0473	3.2811
	Broiler	0.0385	0.0435	0.0335	0.0200	0.0236	0.0163	0.0030	0.0030	0.0030	5.1990	4.1776	6.2203
Eggs and egg products	Egg	0.0326	0.0261	0.0391	0.0143	0.0109	0.0177	0.0030	0.0030	0.0030	5.3328	7.1772	3.4884
	Salted duck egg	0.0262	0.0228	0.0296	0.0193	0.0166	0.0220	0.0030	0.0030	0.0030	3.2390	4.3898	2
Aquatic products	Carp	0.0291	0.0331	0.0251	0.0523	0.0381	0.0664	0.0030	0.0030	0.0030	4.9830	5.5129	4.4531
	Cod	0.0311	0.0321	0.0300	1.4032	1.4272	1.3791	0.0070	0.0073	0.0067	3.4880	3.8000	3.1759
Milk and dairy products	Milk	0.0084	0.0079	0.0102	0.0040	0.0040	0.0040	0.0097	0.0081	0.0112	2.1083	2.9000	1.4000
Vegetables and vegetable products	Turnip	0.0068	0.0111	0.0024	0.0040	0.0040	0.0040	0.0030	0.0030	0.0030	1.9836	3.1898	0.7774
	Beans	0.0088	0.0124	0.0051	0.0040	0.0040	0.0040	0.0030	0.0030	0.0030	3.4082	1.9150	4.9014
	Mungbean sprout	0.0110	0.0155	0.0065	0.0040	0.0040	0.0040	0.0030	0.0030	0.0030	3.1682	4.0672	2.2692
	Eggplant	0.0078	0.0117	0.0040	0.0040	0.0040	0.0040	0.0054	0.0030	0.0078	5.6545	9.6158	1.6932
	Tomato	0.0041	0.0045	0.0037	0.0040	0.0040	0.0040	0.0030	0.0030	0.0030	1.5938	2.4742	0.7134
	Pepper	0.0069	0.0092	0.0047	0.0040	0.0040	0.0040	0.0048	0.0030	0.0066	3.2166	3.3732	3.0600
	Cucumber	0.0025	0.0020	0.0030	0.0040	0.0040	0.0040	0.0030	0.0030	0.0030	3.0849	3.1009	3.0690
	Leek	0.0142	0.0152	0.0132	0.0090	0.0084	0.0096	0.0196	0.0282	0.0111	5.6993	11.0706	0.3281
	Chinese cabbage	0.0029	0.0055	0.0003	0.0040	0.0040	0.0040	0.0071	0.0066	0.0076	5.8179	10.1243	1.5115
	Celery stalk	0.0075	0.0070	0.0081	0.0040	0.0040	0.0040	0.0080	0.0030	0.0129	5.1424	2.8563	7.4286
	Mushroom	0.0070	0.0097	0.0042	0.0228	0.0086	0.0369	0.0397	0.0650	0.0143	6.3841	6.9817	5.7865
	Kelp	0.0394	0.0394	0.0395	1.3468	1.3272	1.3663	0.0183	0.0109	0.0258	5.7764	5.0641	6.4888
	Mustard tuber	0.0296	0.0484	0.0108	0.0068	0.0040	0.0095	0.0123	0.0087	0.0160	4.7712	5.5487	3.9937
Fruits and fruit products	Apple	0.0039	0.0047	0.0031	0.0040	0.0040	0.0040	0.0030	0.0030	0.0030	2.2726	2.8561	1.6892
	Pear	0.0096	0.0096	0.0095	0.0040	0.0040	0.0040	0.0058	0.0086	0.0030	1.7773	1.6972	1.8573
	Peach	0.0087	0.0111	0.0062	0.0040	0.0040	0.0040	0.0067	0.0104	0.0030	1.7155	2.1883	1.2427
	Grape	0.0042	0.0050	0.0035	0.0040	0.0040	0.0040	0.0030	0.0030	0.0030	4.6368	4.2897	4.9839
	Citrus	0.0049	0.0067	0.0030	0.0040	0.0040	0.0040	0.0030	0.0030	0.0030	1.4019	0.8116	1.9921
	Watermelon	0.0019	0.0013	0.0026	0.0040	0.0040	0.0040	0.0030	0.0030	0.0030	1.8480	1.6263	2.0696
Sugar and sugar products	Sugar	0.0056	0.0050	0.0062	0.0040	0.0040	0.0040	0.0030	0.0030	0.0030	10.1962	11.6962	8.6962
Drinking water and beverages	Water	0.0013	0.0013	0.0013	0.0040	0.0040	0.0040	0.0030	0.0030	0.0030	1.8090	2.2097	1.4084
	Orange juice	0.0024	0.0028	0.0020	0.0040	0.0040	0.0040	0.0030	0.0030	0.0030	1.2818	1.5960	0.9676
	Ice bar	0.0038	0.0051	0.0025	0.0040	0.0040	0.0040	0.0030	0.0030	0.0030	1.7702	1.8333	1.7072
Alcoholic drinks	Beer	0.0013	0.0013	0.0013	0.0040	0.0040	0.0040	0.0030	0.0030	0.0030	2.2531	2.6729	1.8333
	Erguotou	0.0013	0.0013	0.0013	0.0040	0.0040	0.0040	0.0030	0.0030	0.0030	4.2643	5.8556	2.6729
In summary		0.0189	0.0199	0.0179	0.0691	0.0682	0.0700	0.0085	0.0080	0.0090	9.3093	9.2044	

**TABLE 3:** Mean concentration of Pb, As, Cd, and Al in different food groups

Food group	Compound			
	Pb	As	Cd	Al
Cereals and cereal products	0.008	0.024	0.004	44.016
Beans, nuts, and their products	0.045	0.020	0.024	3.480
Potatoes and their products	0.061	0.011	0.012	16.424
Meat and its products	0.038	0.018	0.008	4.485
Eggs and egg products	0.029	0.017	0.003	4.286
Aquatic products	0.030	0.728	0.005	4.235
Milk and dairy products	0.007	0.004	0.003	1.984
Vegetables and vegetable products	0.012	0.118	0.011	4.476
Fruits and fruit products	0.006	0.004	0.004	2.361
Sugar and sugar products	0.006	0.004	0.003	10.196
Drinking water and beverages	0.003	0.004	0.003	1.620
Alcoholic drinks	0.001	0.004	0.003	3.259

### 3.2 Food consumption of Jilin Province residents

Table 4 shows that the top three consumed categories in Jilin Province were vegetables and vegetable products, cereals and cereal products, and alcoholic drinks; the mean consumption data of these were 331.35, 307.09, and 226.16 g/day, respectively.

**TABLE 4:** Consumption data of different food groups (g/day)

Food groups	Mean	P50	P90	P95	P97.5
Cereals and cereal products	307.09	250.94	521.35	678.03	892.23
Beans, nuts, and their products	93.39	71.98	185.60	219.62	299.38
Potatoes and their products	81.51	66.67	172.71	217.67	266.67
Meat and its products	68.46	50.65	137.88	176.05	227.46
Eggs and egg products	47.60	39.14	91.68	107.14	135.28
Aquatic products	80.54	66.67	144.22	198.93	213.14
Milk and dairy products	117.95	83.33	224.26	304.25	313.73
Vegetables and vegetable products	331.35	287.49	587.78	734.61	914.74
Fruits and fruit products	159.33	132.46	300.00	381.53	478.81
Sugar and sugar products	20.78	17.86	49.50	49.50	49.50
Drinking water and beverages	106.90	70.23	271.07	386.66	476.00
Alcoholic drinks	226.16	166.67	588.05	757.83	808.44

### 3.3 Exposure to the four heavy metals in the total diet of residents of different age‐groups

Table 5 presents the mean exposure level of the four heavy metals in different age‐groups of Jilin Province residents. Table 4 shows that exposure to the four heavy metals decreases with age; the 2–6 age‐group had the highest exposure among the whole population. The average exposure to the four heavy metals among different age‐groups was lower than their corresponding health guidance value or benchmark dose. However, the MOE of Pb in the overall population was 3.71, and the exposure to Al accounted for 48.61% of PTWI. For the 2–6 age‐group, the Pb exposure level was 0.51 μg/kg BW, and the MOE was 1.18, close to 1. The exposure level to Al in the 2–6 age‐group was 204.96 μg/kg BW, which accounted for 71.74% of PTWI; thus, the exposure level is close to the PTWI.

**TABLE 5:** Mean exposure to the four heavy metals from total diet in different age‐groups and comparison with their health guidance values or benchmark doses

Population group	Number of people	Pb		As		Cd		Al	
		Exposure, μg/kg·bw	MOE	Exposure , μg/kg·bw	MOE	Exposure , μg/kg·bw	PTMI%	Exposure , μg/kg·bw	PTWI%
2–6 years	87	0.51	1.18	0.52	5.72	0.22	26.52	204.96	71.74
7–17 years	450	0.34	3.53	0.39	7.69	0.13	15.10	162.03	56.71
18–40 years	1774	0.32	3.73	0.33	9.19	0.11	12.99	119.25	41.74
41–65 years	4,146	0.19	6.26	0.25	11.87	0.08	9.89	113.60	39.76
>65 years	1,243	0.26	4.66	0.26	11.71	0.07	8.70	94.62	33.12
Overall population	7,700	0.32	3.71	0.35	8.57	0.12	14.64	138.89	48.61

### 3.4 Contribution rate analysis of exposure to the four heavy metals from different food groups

**FIGURE 1:**
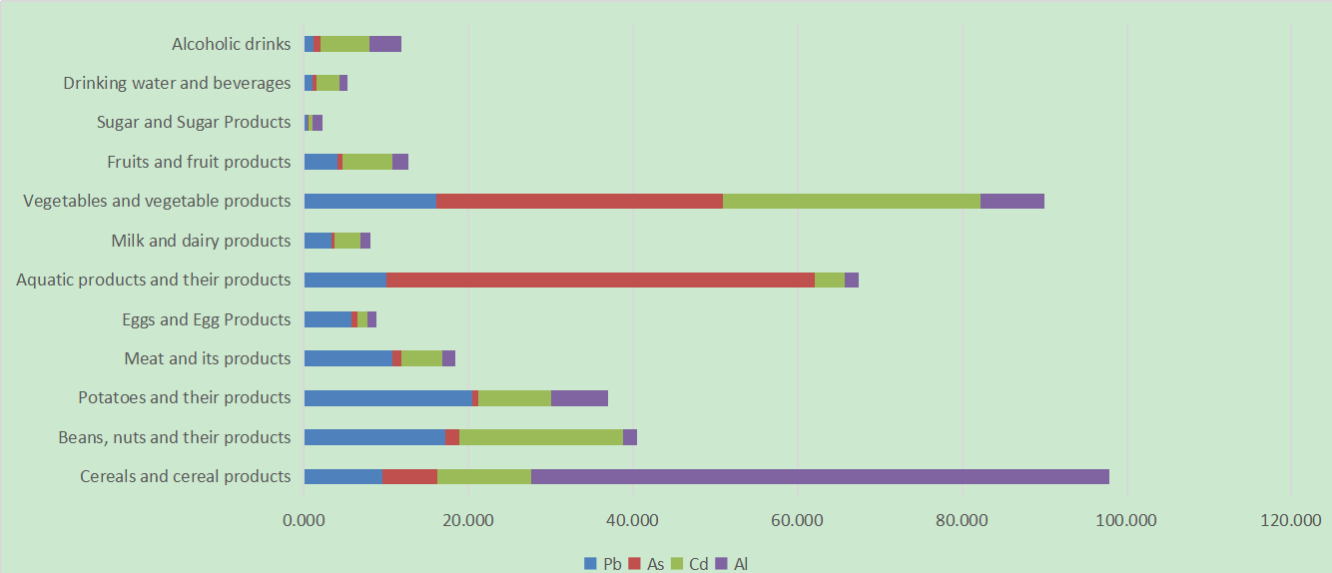
Exposure contribution rate of different food groups to the four heavy metals

Table 6 shows the mean exposure of Pb, As, Cd, and Al from different food groups. The contribution rate to total dietary exposure is shown in Figure 1. For Pb, potatoes and their products were the main source and the contribution rate was 24.89%, followed by beans, nuts, and their products, for which the contribution rate was 17.21%. For As, more than half of the As exposure was contributed by aquatic products and their derivatives (52.14%), followed by vegetables and vegetable products (34.82%). For Cd, the highest two contribution rates were vegetables and vegetable products (31.3%), and beans, nuts, and their products (19.97%). For Al, the main source was cereals and cereal products; the contribution rate was as high as 70.22%, followed by vegetables and vegetable products, which accounted for 7.71%.

**TABLE 6:** Mean exposure of the four heavy metals from different food products

Food group	Compound			
	Pb	As	Cd	Al
Cereals and cereal products	0.039	0.124	0.021	225.282
Beans, nuts, and their products	0.070	0.031	0.037	5.417
Potatoes and their products	0.083	0.015	0.016	22.310
Meat and its products	0.044	0.020	0.009	5.118
Eggs and egg products	0.023	0.013	0.002	3.400
Aquatic products	0.040	0.977	0.007	5.685
Milk and dairy products	0.013	0.008	0.006	3.899
Vegetables and vegetable products	0.065	0.652	0.059	24.721
Fruits and fruit products	0.017	0.011	0.011	6.269
Sugar and sugar products	0.002	0.001	0.001	3.531
Drinking water and beverages	0.004	0.007	0.005	2.887
Alcoholic drinks	0.005	0.015	0.011	12.283

## 4 DISCUSSION

The FAO/WHO recommended three dietary exposure methods, including TDS, selective study of individual foodstuffs, and duplicate portion study. Each method has its own merits and demerits. Selective study of individual foodstuffs is for a representative food, not for the whole population, and is generally used for preliminary assessments. Although the duplicate portion study is the most accurate, it is difficult to carry out on a large scale because of its heavy workload and high cost. TDS, also known as market basket survey, can obtain the intake of food pollutants in a population or subpopulation by directly detecting the contents of all kinds of pollutants in the diet (including drinking water) of residents after cooking. The measured food samples were close to the actual consumption state of residents. TDS is currently internationally recognized to evaluate a country or a subpopulation. It is a general and optimal method for measuring the intake of chemical pollutants in the diets of large‐scale populations (Joint FAO & WHO, 1985; World Health Organization, 2005). Jilin Province took part in the sixth national TDS; this was second time this region has taken part in a TDS. But this was the first time that we estimated the principal heavy metal exposure in the residents of Jilin Province. Furthermore, we expanded the sampling points to six places, last time we only had three. We wanted to study whether heavy metal pollution in the Songhua River Basin was more serious than in other areas. The results of this study showed significant differences in the two areas. We can deduce that industrial bases in the Songhua River Basin did not affect residents' exposure to heavy metals.

According to the availability of data on food consumption and compound content, three evaluation models can be constructed, including point estimate models (EFSA Journal, 2007b), simple distribution models (Lambe, 2002), and probability models (McNamara, Naddy, Rohan, & Sexton, 2003; Zartarian, Glen, Smith, & Xue, 2008). These three methods have their own advantages and disadvantages. Simple distribution models, which take into account the distribution of consumption, are particularly applicable to comparisons of exposure in countries and regions with different consumption patterns. Probability models use quantitative information on consumption and chemical data to quantify the variability and uncertainty of exposures, resulting in a fine assessment, but require large resources and are particularly cumbersome to calculate. Point estimate models are simple to construct and highly conservative and are suitable for chemical screening studies (Lambe, 2002). Because this was the first time we have estimated the principal heavy metal exposure in the residents of Jilin Province, we used point estimate models, with the aim of preliminarily screening the exposure risk.

The studied heavy metal contamination level in Jilin Province was relatively light. The mean concentration of Cd in all foodstuffs of the total dietary intake in 2000 was 0.046 mg/kg (Zhang et al., 2008). The mean concentration of Cd in this study was 0.0085 mg/kg. Milk and dairy products were the lowest Pb contaminated foods in Jiangsu Province in 2014; the mean concentration was 0.036 mg/kg (Jin et al., 2014), which was higher than the average Pb content (0.0189 mg/kg) in all foodstuffs found in this study. The highest content of As in this study was cod, with a mean concentration of 1.403 mg/kg. However, a previous study showed that inorganic As accounts for 2%–4% of the total arsenic in aquatic products (Joint FAO/WHO Expert Committee on Food Additives & WHO Organization, 2011a). Therefore, if we only analyze inorganic As, the contamination level is dramatically decreased and the risk is low. The Al content in fried sticks was more than twice the MAC of the national standard. It may be that there were some problems in the use of food additives containing Al in producing fried sticks. The contamination of Al in fried food needs further consideration.

Dietary exposure risk assessment revealed that the exposures to Cd and Al in the whole population were lower than the corresponding health guidance value, and the MOE for Pb and inorganic As was higher than 1. However, the MOE of Pb in the 2–6 age‐group was 1.18, close to 1. The exposure level to Al in the 2–6 age‐group accounted for 71.74% of PTWI; thus, the exposure level was close to the PTWI. Because children's organs are more sensitive to heavy metal pollution than adults, more attention should be paid to children aged 2–6 years. Potatoes and their products were the main sources of dietary exposure to Pb. Aquatic products and their derivatives had the highest contribution to As exposure. Vegetables and vegetable products were the main sources of dietary exposure to Cd. Al in the diet was primarily contributed by cereals and cereal products, which accounted for 77.22% of Al exposure. That was because of the high content and high consumption of cereals and cereal products; thus, the high‐consuming population may need to pay attention to this exposure.

A preliminary assessment of exposure to four heavy metals in residents of Jilin Province was made in this study. However, there were some uncertainties, and more accurate assessments are needed for the high consumption population. Moreover, a cumulative exposure assessment of heavy metals needs to be considered, making sure the cumulative exposure risk of heavy metals in foods is safe. The results of this study put forward an extensive concern for residents of Jilin Province, especially for children aged 2–6 years. In summary, more effort is needed to continue to reduce heavy metal exposure from all sources.
